# Toward Stable Li‐Mediated Nitrogen Reduction: Strategies, Milestones, and Future Outlook

**DOI:** 10.1002/cssc.202500674

**Published:** 2025-09-23

**Authors:** Jinwoo Chu, Sungbin Yang, Byungha Shin

**Affiliations:** ^1^ Department of Materials Science and Engineering Korea Advanced Institute of Science and Technology (KAIST) Daejeon 34141 Republic of Korea

**Keywords:** ammonia production, electrocatalysis, Li‐mediated nitrogen reduction, nitrogen fixation, stability

## Abstract

Li‐mediated nitrogen reduction reaction (Li‐NRR) has emerged as a promising alternative to the traditional Haber–Bosch process for ammonia synthesis, offering the potential for sustainable and energy‐efficient production under ambient conditions. Over the past 5 years, Li‐NRR research has grown significantly, leading to rapid advancements in ammonia production rates and Faradaic efficiency. This review focuses on the recent advancements in Li‐NRR, with a particular emphasis on strategies aimed at improving system stability. Various strategies to enhance the stability of Li‐NRR systems are categorized and analyzed, including the optimization of proton carriers, the design of favorable solid‐electrolyte interphases, and the regulation of anodic reactions to prevent electrolyte decomposition. The review also highlights the growing importance of materials selection and reaction conditions, such as electrolyte composition and electrode design, in achieving high Li‐NRR system stability. Despite rapid progress, several current challenges are identified, and future directions for research to create more robust and sustainable Li‐NRR systems are proposed. This work aims to provide insights into the critical factors that drive the performance and stability of Li‐NRR and to inspire future efforts toward the development of efficient and scalable ammonia synthesis technologies.

## Graphical abstract

This review spotlights Li‐mediated nitrogen reduction strategies for enhancing stability. Through insights into various proton carriers, favorable solid‐electrolyte interface design, anodic control, and other strategies, it paves the way for sustainable, decentralized green ammonia production.

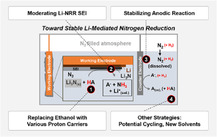

## 1 Introduction

Ammonia is one of the most extensively used chemical compounds worldwide, serving as a foundational feedstock in agriculture for fertilizers and functioning as a crucial raw material in diverse industries—including pharmaceuticals, plastics, and explosives.^[^ ^1^ ^]^ Additionally, because of its remarkably high volumetric and gravimetric hydrogen density, ammonia stands out as a promising candidate among carbon‐free hydrogen storage materials.^[^ ^2^, ^3^ ^]^ Accordingly, ammonia is well‐positioned to remain indispensable, beyond its well‐established and current uses, in a future likely to see an expanded reliance on hydrogen as a primary energy vector.

Compared with other commonly used industrial chemicals, ammonia exhibits the highest production volume, energy demand, and associated carbon dioxide emissions, resulting in one of the most substantial environmental footprints among bulk chemicals.^[^ ^4^ ^]^ Each year, 175 megatons of ammonia are produced, accounting for 1.8% of global energy consumption and 1.4–1.8% of worldwide CO_2_ emissions.^[^ ^5^, ^6^ ^]^ This is primary because ammonia is still predominantly synthesized via the Haber–Bosch process, the only commercially viable method to date.

In the Haber–Bosch process, pure hydrogen and nitrogen are reacted in dedicated industrial plants under high‐temperature (350–500 °C) and high‐pressure (150–300 bar) conditions.^[^ ^5^ ^]^ To reach the required high temperature and pressure, fossil fuels are burned for heating, and steam methane reforming is used to supply pure hydrogen, which leads to significant CO_2_ emissions. In addition, the large scale of these Haber–Bosch plants necessitates centralized production facilities, which in turn increases logistical costs for distributing ammonia to the diverse end users that require it.^[^ ^7^ ^]^

As a strategy to address the environmental and transportation challenges posed by the Haber–Bosch process, electrochemical nitrogen reduction (ENRR) and electrochemical nitrate reduction (E‐NO_3_RR) to ammonia has been proposed. These methods avoid the direct use of hydrogen gas by instead supplying hydrogen through hydronium or hydroxide ions in aqueous solutions or via hydrogen‐ion carriers in organic electrolytes, thereby eliminating CO_2_ emissions during production. Furthermore, because these reactions occur under low temperature and pressure, there is no need for fossil‐fuel‐based heating or large‐scale plants to maintain high‐temperature, high‐pressure conditions.^[^ ^7^, ^8^, ^9^, ^10^ ^]^ As a result, these routes are more sustainable and enables small‐scale, decentralized ammonia production systems.

In the case of E‐NO_3_RR, the reaction has also been investigated for environmental nitrate mitigation and wastewater treatment through strategies such as electrocatalysis, photocatalysis, and photo‐Fenton processes.^[^ ^11^, ^12^, ^13^, ^14^ ^]^ Compared with ENRR, E‐NO_3_RR is thermodynamically more favorable, offering the advantages of relatively higher ammonia yield rates and Faradaic efficiencies. However, in terms of serving as a substitute for the Haber–Bosch process, E‐NO_3_RR faces certain challenges, as the concentration of nitrate available in waste is much lower than that of nitrogen gas and the separation of the as‐synthesized ammonia from the reaction electrolyte remains difficult.^[^ ^11^, ^15^ ^]^

ENRR can be broadly categorized into two approaches: 1) direct ENRR in aqueous media (aqueous‐ENRR) and 2) Li‐mediated nitrogen reduction (Li‐NRR). In aqueous‐ENRR, molecular nitrogen is directly cleaved at the catalyst surface in an aqueous electrolyte and subsequently hydrogenated to form ammonia. This reaction proceeds under ambient temperature and pressure, and in principle the theoretical maximum energy efficiency (82.8%) can match that of a water‐splitting‐based Haber–Bosch route.^[^ ^16^ ^]^ However, a critical drawback is that most catalysts share active sites for both ammonia formation and the competing hydrogen evolution reaction (HER), leading to significantly reduced Faradaic efficiency and/or ammonia production rates.^[^ ^16^, ^17^, ^18^, ^19^ ^]^

**Figure 1:**
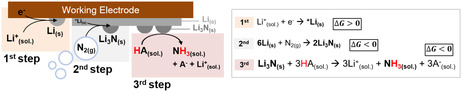
Schematic of the Li‐NRR. (*g*) indicates a gas, (*s*) indicates a solid, and (sol.) indicates a solution. Dissolved N_2_ gas is shown as N_2(g)_ bubble in the scheme. HA(sol.) represents the proton carrier that contains hydrogen, which can be released to Li_3_N to induce ammonia synthesis.

By contrast, Li‐NRR does not electrochemically split dinitrogen at the electrode surface. Instead, Li ions (dissolved in an organic electrolyte such as tetrahydrofuran (THF), along with a suitable proton carrier) are electrochemically reduced to Li metal on the working electrode surface (see **Figure** **1**). Owing to its high energy state, the freshly deposited Li metal spontaneously reacts with N_2_ to form lithium nitride, which then undergoes further spontaneous chemical reactions with the proton carrier to generate ammonia.^[^ ^20^, ^21^, ^22^ ^]^ Although the maximum theoretical energy efficiency of this approach (about 28%) is lower than modern Haber–Bosch plants (around 56–62%), Li‐NRR offers several advantages.^[^ ^16^, ^23^ ^]^ It typically achieves higher Faradaic efficiencies and higher ammonia production rates because it is less prone to competing HER under these reaction conditions, and the organic, aprotic electrolytes commonly used offer greater N_2_ solubility compared to aqueous systems. As a result, Li‐NRR research has gained momentum over the past 5 to 10 years, paralleling the burgeoning interest in aqueous‐ENRR.^[^ ^24^, ^25^, ^26^, ^27^, ^28^, ^29^, ^30^, ^31^, ^32^, ^33^ ^]^

Following the first report of NRR by Fichtner et al. in 1930 and the initial comparison of Li‐NRR catalysts and materials by Tsuneto et al. in 1993, subsequent developments—ranging from potential cycling methods and the achievement of nearly 100% Faradaic efficiency to continuous‐flow ammonia synthesis—have significantly advanced performance, with high Faradaic efficiencies and ammonia production rates.^[^ ^21^, ^34^, ^35^, ^36^, ^37^, ^38^, ^39^ ^]^ In addition to boosting performance, improving system stability has emerged as a major focus of Li‐NRR research. Early studies often lacked detailed stability assessments, and many early reports evaluated yield and efficiency only over short (≈1 h) reaction periods. However, with the advent of new strategies, continuous chronoamperometry tests extending to around 93 h stable operation and potential cycling or flow‐reactor‐based operation lasting up to ≈300 h have been achieved, marking substantial progress in prolonging operational stability.^[^ ^40^, ^41^ ^]^ Beyond these advances, studies on analytical methods (as noted in the Conclusion) and methodological approaches(e.g., multivariate approaches) for better understanding Li‐NRR have also been undertaken, contributing to the broader effort toward achieving high‐efficiency and long‐term stable Li‐NRR systems.^[^ ^42^ ^]^

This review highlights various efforts aimed at enhancing the stability of Li‐NRR systems. Owing to the inherent interdependence of efficiency and stability in electrochemical processes, most stability‐focused approaches also tend to improve Li‐NRR performance overall. Hence, we discuss strategies devised to realize more robust Li‐NRR, emphasizing the specific discoveries and accomplishments that have contributed to stability improvements. These strategies are categorized into three main directions: 1) selecting a suitable proton carrier to stabilize operation, 2) tuning the solid–electrolyte interphase (SEI) properties through additives, and 3) controlling the anodic reaction to enhance overall cell stability. In each category, we highlight key milestones and then discuss current challenges and propose future directions for continued advancements in Li‐mediated NRR in the conclusion.

## 2 Replacing Ethanol with Various Proton Carriers in Li‐NRR

In Li‐NRR, the proton carrier supplies hydrogen ions to lithium nitride, enabling spontaneous chemical reactions that yield ammonia. Consequently, it must both effectively deliver hydrogen and remain stable under the aprotic, high‐potential (≈3 V) conditions of Li‐NRR. After study by Tsuneto et al. introduced ethanol as a proton carrier, most early Li‐NRR research also used ethanol.^[^ ^18^, ^21^, ^22^, ^35^, ^36^, ^37^, ^43^ ^]^ Upon reacting with lithium nitride (Li_3_N), ethanol releases hydrogen and leaves behind oxidized ethoxide (EtO^−^). While the exact behavior of this EtO^−^ remains unclear, MacFarlane et al. have proposed that EtO^−^ can further react with THF to form 2‐ethoxytetrahydrofuran as a side reaction, whereas Steinberg et al. used cryo‐electron microscopy (cryo‐EM) analyses to suggest that ethoxide‐related species (e.g., LiOEt) could accumulate on the SEI surface.^[^ ^44^, ^45^ ^]^

Early Li‐NRR studies typically did not assess long‐term stability beyond brief (≈1 h) chronoamperometry or chronopotentiometry measurements aimed at demonstrating Faradaic efficiency. As interest grew in improving the stability of Li‐NRR systems, ethanol intrinsic reactivity emerged as a key factor potentially limiting stability. Because ethanol is highly reactive at ≈3 V, it may undergo unwanted oxidation pathways even before serving its intended role as a proton donor; moreover, the resulting EtO^−^ can itself lead to further side reactions at the anode.^[^ ^44^ ^]^ Consequently, researchers have explored various alternative proton carriers to ascertain whether these substitutions could enhance both the performance and stability of Li‐NRR systems.^[^ ^40^, ^44^, ^46^, ^47^, ^48^, ^49^, ^50^, ^51^, ^52^ ^]^

### 2.1 Replacing Ethanol with Different Alcohols

**Figure 2:**
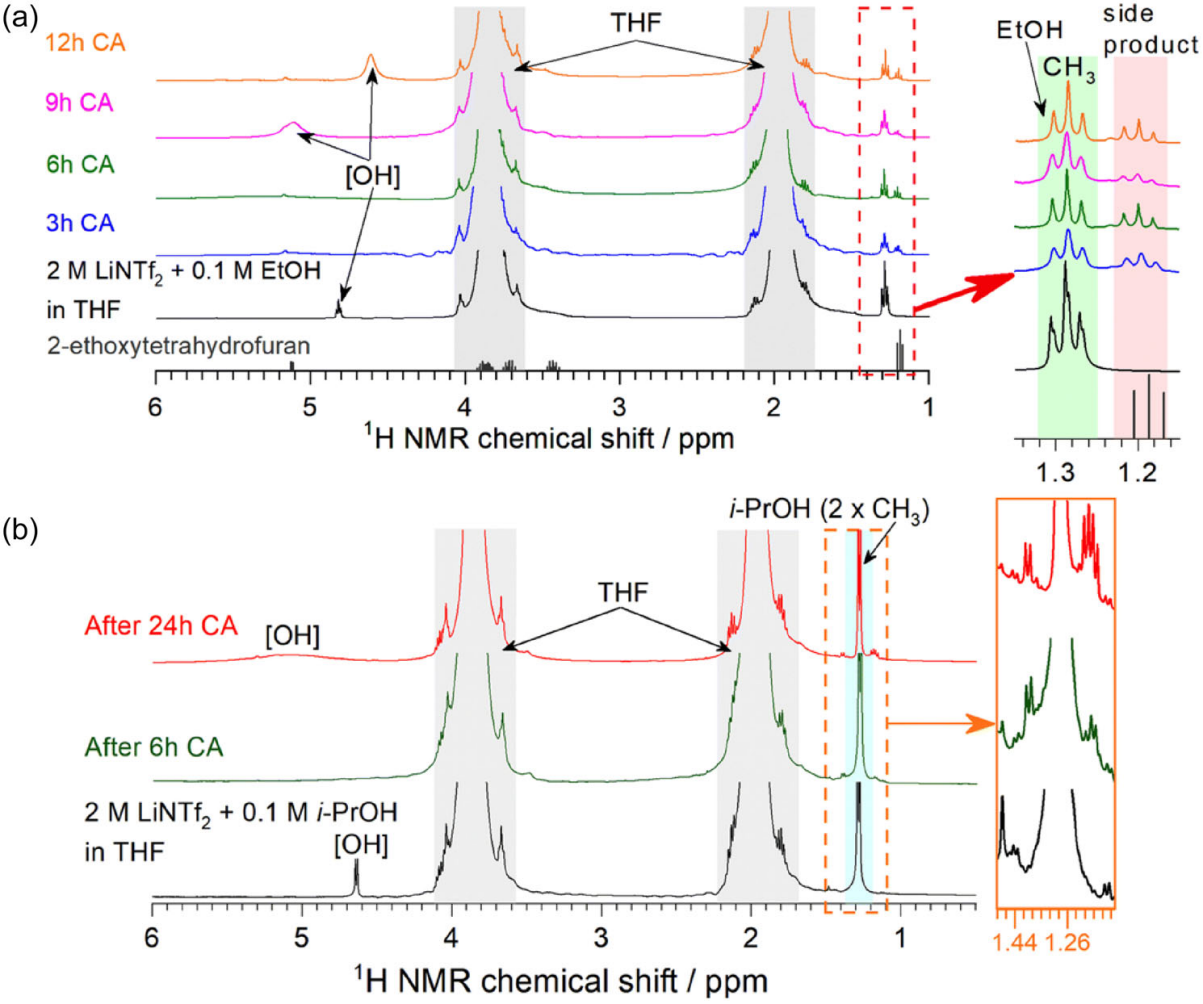
^1^H NMR spectra of EtOH and i‐PrOH based electrolyte before and after Li‐NRR reaction. a) Spectra of the electrolyte with EtOH and b) with i‐PrOH as proton carriers. The red box indicates the ethanol‐derived side product after the reaction. Orange square highlights changes in the side product after 6 and 24 h of chronoamperometry. Adapted with permission from ref. [44]. Copyright 2023, Royal Society of Chemistry.

Du et al. examined a wide array of proton carriers (various alcohols including ethanol, a phosphonium proton shuttle, water, and THF) to compare their performances in Li‐NRR.^[^ ^44^ ^]^ Among the candidates tested, ethanol achieved the highest ammonia yield. However, the authors noted pronounced color changes in the electrolyte when ethanol was used, attributing this phenomenon to the electrooxidation processes linked to both ethanol and tetrahydrofuran. To identify likely side products, they performed ^1^H‐nuclear magnetic resonance(^1^H‐NMR) and ^13^C‐NMR analyses of the electrolyte; based on characteristic peaks observed in the ^1^H‐NMR spectra, 2‐ethoxytetrahydrofuran and pentanal diethyl acetal were proposed as plausible byproducts (see red box in **Figure** **2**a). They further showed that, when using propan‐2‐ol (i‐PrOH) or propan‐1‐ol (n‐PrOH), the intensities of these side‐product NMR peaks were significantly reduced (Figure 2b). Notably, i‐PrOH delivered a Faradaic efficiency of 96%—nearly matching with ethanol (98%)—and was thus proposed as a more stable proton carrier alternative. In 24 h tests, i‐PrOH achieved high Faradaic efficiency (96%) without performance degradation. Although longer‐term results were not reported, the authors suggested that i‐PrOH may enable stability comparable to or better than ethanol, for which 90 h stable operation has been documented under analogous conditions in separate studies.

Krishnamurthy et al. systematically explored an array of alcohol‐based proton carriers as potential alternatives to ethanol, aiming to elucidate correlations between their chemical properties and Li‐NRR performance.^[^ ^46^ ^]^ They focused on Kamlet–Taft (KT) parameters, using a deep‐learning model trained on known KT values to predict the *α* (proton‐donating) and *β* (proton‐accepting) parameters of alcohols not yet experimentally characterized. Their findings suggested that effective Li‐NRR proton carriers typically satisfy *α*  >  0.78 and *β*  >  0.57. However, other reports have challenged the direct correlation to these KT parameters, asserting that additional factors beyond *α* and *β* may be at play and warrant further investigation.

In a subsequent study, Lazouski et al. examined how different alcohols affect SEI formation and Li‐NRR performance.^[^ ^47^ ^]^ They confirmed that neither diffusivity nor pK_a_ strongly correlates with Li‐NRR activity; instead, even among proton carriers with similar chemical properties, structural differences exert a more pronounced influence on ammonia production. Building on this observation, they proposed a model linking the diffusion rates of N_2_, the proton carrier, and ammonia to Faradaic efficiency, suggesting that proton‐carrier concentration modulates SEI permeability and thereby influences optimal performance. Although they observed that 1‐butanol offered superior performance compared to ethanol, they emphasized the need for further exploration of anode stability and recyclability before proposing it as a good alternative. Although their two studies did not extensively address long‐term stability, the significance of this study lies in identifying 1‐butanol and 1‐propanol, among the diverse alcohol‐based proton carriers screened, as particularly promising for further investigation.

### 2.2 Replacing Ethanol with Ionic Liquids

Building on efforts to identify alternatives to alcohol‐based proton carriers for Li‐mediated NRR, several studies have explored completely different classes of compounds for Li‐NRR proton carriers that offer both high performance and improved stability. In 2021, Suryanto et al. reported using a phosphonium‐based proton carrier, trihexyltetradecylphosphonium cation ([P_6,6,6,14_]^+^).^[^ ^40^ ^]^ When this proton carrier reacted with lithium nitride, an adjacent carbon on the phosphonium lost a hydrogen, yielding a phosphonium ylide; upon the addition of a mild acid, hydrogen was replenished and the carrier was effectively recycled, as confirmed by NMR analysis. Under a 19.5 bar N_2_/0.5 bar H_2_ mixture (without stirring), they achieved a remarkable Faradaic efficiency of 69% maintained stably over 20 h. Although stirring the electrolyte led to a slightly lower efficiency of 45%, it remained stable for 93 h: one of the longest reported durations for Li‐NRR with consistent ammonia yield and Faradaic efficiency. This work demonstrated the potential of ionic liquids as proton carriers for enhancing Li‐NRR stability.

Inspired by the above findings, subsequent studies emerged using ionic liquids to achieve exceptionally stable Li‐NRR performance. For instance, Nguyen et al. investigated various phosphonium‐based proton carriers differing in alkyl‐chain length and compared their Li‐NRR activities.^[^ ^48^ ^]^ Under optimal conditions using 2 M bis(trifluoromethane)sulfonimide lithium salt(LiNTf_2_) + 0.1 M [P_6,6,6,14_]^+^, the system maintained stable operation for 72 h. In addition to analyzing diffusion properties of Li^+^ and proton carriers and associated SEI composition changes with varying chain lengths, these authors reported a new insight into electrolyte stability: although THF oxidation can increase solution viscosity and impede mass transport, it does not necessarily degrade Faradaic efficiency, indicating that extensive THF oxidation need not compromise Li‐NRR performance. However, electrode arrangement significantly affected outcomes, highlighting the need for further investigation.

**Figure 3:**
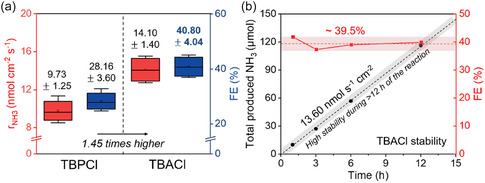
Li‐NRR with tetraalkyl‐type ionic liquids (TBPCl and TBACl). a) Comparison of Li‐NRR performance between TBPCl and TBACl. b) Stable ammonia production (black symbols) and the Faradaic efficiency (red symbols) with TBACl as proton carrier for 12 h experiments. Adapted with permission from ref. [49]. Copyright 2024, Wiley‐VCH GmbH.

Meanwhile, Yang et al. compared phosphonium‐ and ammonium‐based ionic liquids as proton carriers for Li‐NRR and performed stability tests.^[^ ^49^ ^]^ They chose tetrabutylphosphonium chloride (TBPCl) and tetrabutylammonium chloride (TBACl) to isolate the effect of the central atom, while keeping the alkyl chains and anion identical. While scanning electron microscope (SEM) and X‐ray photoelectron spectroscopy (XPS) analyses showed only minor differences in the SEI formed by TBPCl and TBACl, they proposed that variations in pK_a_ largely account for the discrepancy in Li‐NRR performance: under these conditions, a lower pK_a_ allows more time for Li to react with N_2_ (forming Li_3_N) before hydrogen donation occurs, thereby increasing overall efficiency (**Figure** **3**a). With the best‐performing condition with TBACl, they exhibited stable ammonia production and improved Faradaic efficiency (39.5%) over a 12 h period (Figure 3b). Collectively, these ionic‐liquid studies stand out for demonstrating high stability not only in terms of working‐electrode potential and Faradaic efficiency but also in maintaining steady ammonia production rates. This contrasts with many ethanol‐based systems, where electrode stability is often reported separately, but ammonia yield can decline over time. Given these encouraging results, continued exploration of ionic‐liquid proton carriers seems poised to yield notable advances in Li‐NRR.

### 2.3 Replacing Ethanol with Other H‐Containing Materials

**Figure 4:**
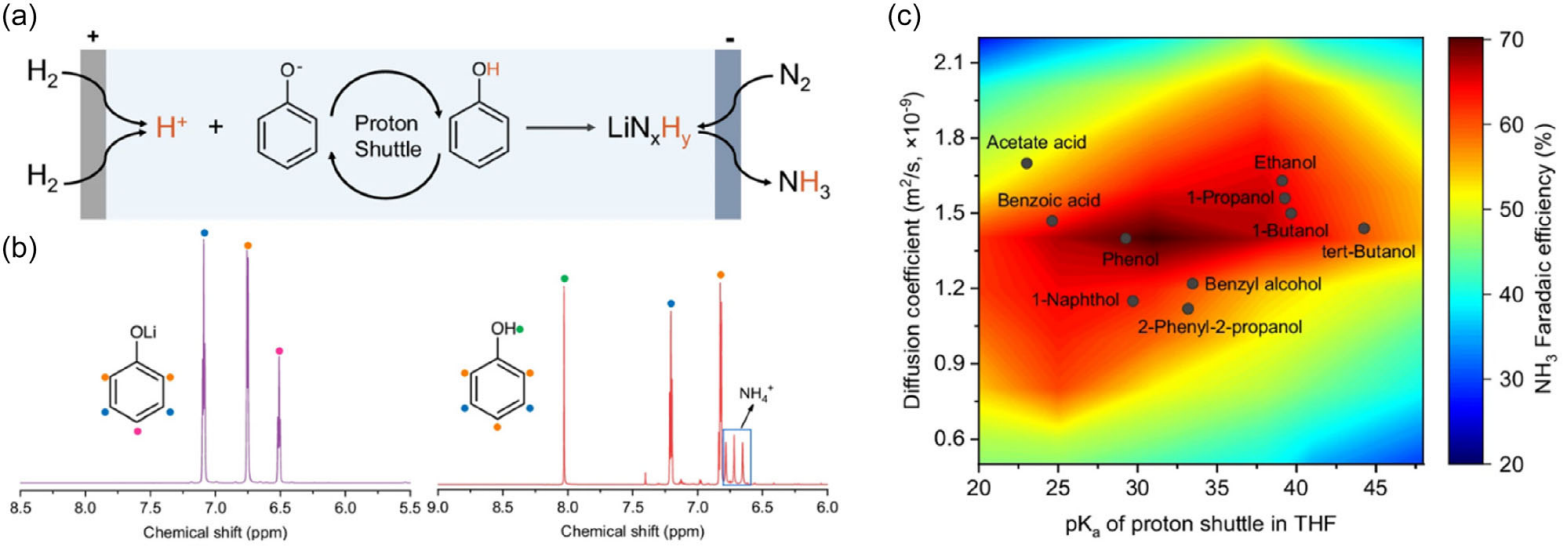
Continuous‐flow Li‐NRR with phenol as a proton carrier. a) Schematic of the Li‐NRR process in a flow cell via proton shuttling by phenol. b) ^1^H NMR spectra of the electrolyte using lithium phenoxide before and after the Li‐NRR chronopotentiometry. c) Heatmap of the predicted ammonia Faradaic efficiency as a function of the pK_a_ of the proton carrier in THF and diffusion coefficient. Adapted with permission from ref. [52]. Copyright 2024, Springer Nature.

Beyond alcohols and ionic liquids, several other compounds—phenols, amines, or even THF itself—have been explored as potential proton carriers for Li‐NRR, with some showing excellent performance and stability.^[^ ^50^, ^51^, ^52^ ^]^ In 2024, Fu et al. used phenol in a gas diffusion electrode–based continuous‐flow Li‐NRR reactor, observing higher performance and greater stability compared with ethanol (**Figure** **4**a).^[^ ^52^ ^]^ The authors attributed this improvement to the resonance‐stabilized phenolate ion (PhO^−^), which is significantly more stable than ethoxide (EtO^−^) and thus confers enhanced chemical and electrochemical resilience. Moreover, NMR analyses revealed that the amount of phenol remained virtually unchanged following the ammonia‐synthesis reactions (see Figure 4b), indicating that phenol was not consumed by side reactions and underscoring its robust stability. Beyond their experimental findings, Fu et al. performed density functional theory (DFT) calculations on various compounds (alcohols, amines, phenols, acids, and phosphonium salts), concluding that both the pK_a_ and the diffusion coefficient of a proton carrier influence ammonia Faradaic efficiency (Figure 4c). Their computational and experimental results consistently identified phenol as the most suitable and stable proton carrier. Given that a previous study using ethanol in the same system reported stable operation only for about 10 h, phenol appears poised to enable much longer continuous operation. If future long‐term stability tests confirm these predictions, phenol could emerge as a highly promising alternative to ethanol in Li‐NRR systems.

## 3 Moderating Li‐NRR SEI Composition and Properties with Additives

In Li‐based batteries and Li‐mediated NRR, one of the most critical considerations—unlike in many other electrocatalytic processes—is the formation of a SEI on the catalyst surface. Under the high‐voltage conditions characteristic of lithium‐based electrochemical reactions, substantial electrolyte decomposition occurs at the Li‐NRR working electrode, leading to the formation of a thick SEI. This interphase is consisted of reduced aprotic solvents (primarily THF), byproducts of the Li precursor, and proton carriers, which are either electrochemically reduced at the electrode surface or undergo chemical reduction by highly reactive Li metal, generating Li composites (e.g., Li_2_O, LiF, Li_2_CO_3_, or LiOEt, depending on the precursor and reactions with ethanol).^[^ ^45^, ^53^ ^]^

Key SEI properties such as thickness, composition, and porosity strongly influence the diffusion rates of Li^+^, N_2_, and the proton carrier.^[^ ^37^ ^]^ When these rates are appropriately balanced, side reactions with Li metal are minimized, enabling selective formation of Li_3_N and, ultimately, high ammonia yields. A uniformly deposited SEI also prevents unwanted dendrite growth, thereby reducing further electrolyte decomposition by Li metal and maintaining a stable steady‐state environment for long‐term operation.^[^ ^54^, ^55^, ^56^ ^]^

Because the SEI characteristics are almost entirely determined by the electrolyte composition and experimental setup (including cell design and stirring conditions), researchers have systematically explored various Li precursors and proton carriers to identify conditions that yield an optimal SEI that minimizes side reactions with favorable ion and chemical diffusion rates, and develops in a stable, uniform manner, leading to steadily improving efficiencies. More recently, to enhance both performance and overall system stability, organic and inorganic additives have been introduced to intentionally tailor SEI properties and create more favorable reaction conditions near the working electrode.^[^ ^55^, ^57^, ^58^, ^59^, ^60^, ^61^, ^62^, ^63^ ^]^ In particular, the studies introduced in the following sections go beyond reporting performance alone, using extensive SEI characterization (e.g., X‐ray diffraction (XRD), XPS, SEM, time‐of‐flight secondary ion mass spectrometer (TOF‐SIMS), NMR) combined with computational studies (such as DFT) to illuminate the mechanisms of SEI formation and guide the development of morphologies and compositions that promote stable, high‐efficiency ammonia synthesis.

### 3.1 Effect of Atmospheric Components Addition on Li‐NRR Stability

In 2021, Li et al. demonstrated a breakthrough in Li‐NRR performance and stability by introducing a small amount of O_2_ alongside N_2_ in the feed gas.^[^ ^55^ ^]^ Specifically, adding 0.8 mol% O_2_ to 20 bar of N_2_ yielded a Faradaic efficiency of ≈78%. Chronopotentiometry measurements further revealed that higher O_2_ concentrations stabilized the working electrode potential for over 2 h, although an excessive O_2_ fraction shifted the system toward oxygen reduction reactions (ORRs) and reduced efficiency. The authors attributed these effects to changes in SEI composition, explaining that a more homogeneous SEI led to uniform Li plating and thus enhanced cell‐potential stability. Additionally, the reduced Li diffusion rate conferred by the modified SEI allowed to reach higher Faradaic efficiencies according to their previously proposed microkinetic model, which holds that increasing the relative diffusion rates of proton and nitrogen compared to Li (r_H_/r_Li_ and r_N2_/r_Li_) prevents side reactions caused by excessive Li plating. XRD analyses confirmed that Li_3_N was abundant at the optimal O_2_ concentration (0.8 mol%), and XPS analysis showed further increases in O_2_ produced oxygen‐derived SEI components such as Li_2_O and LiOH, indicating that O_2_ systematically altered the SEI composition in ways favorable to Li‐NRR. Follow‐up studies using gas chromatography‐mass spectrometry (GC–MS) and NMR showed that increasing O_2_ content suppressed electrolyte decomposition and reduced the formation of byproducts (e.g., polytetrahydrofuran), further supporting the conclusion that O_2_ addition boosts both Li‐NRR efficiency and chemical stability.^[^ ^57^ ^]^

Spry et al. proposed that water concentration significantly influences Li‐NRR efficiency and SEI properties.^[^ ^58^ ^]^ Although water is typically considered detrimental, favoring hydrogen evolution at the expense of ammonia formation, they observed that a small amount (≈36 mM) of water actually improved Li‐NRR efficiency. Post‐reaction XPS analyses of the working‐electrode SEI revealed that water addition lowered the relative amounts of LiCl and LiClO_*n*_, while increasing Li_2_O content—a trend aligning with Li et al.'s findings. Although Spry et al. did not explicitly report stability tests, these studies collectively suggest that both O_2_ and H_2_O can steer the SEI composition toward an oxide‐rich layer, potentially reducing Li^+^ diffusion and enhancing the overall performance and stability of Li‐NRR systems.

### 3.2 Incorporation of Inorganic Additives in Li‐NRR

Jeon and colleagues introduced a small quantity of the silver precursor AgTFSI into a lithium bis(trifluoromethanesulfonyl)imide (LiTFSI)‐based electrolyte, prompting the formation of Ag–Li alloys during Li deposition and SEI formation—an approach that boosted both Li‐NRR efficiency and stability.^[^ ^59^ ^]^ XPS measurements revealed that introducing Ag into electrolytes containing fluorine‐based Li precursors (LiTFSI, LiBF_4_, LiOTf, etc.) led to an LiF‐enriched SEI formation on the working electrode, a result consistent with previous findings that LiF plays a crucial role in constructing a stable SEI. Indeed, the silver‐containing system delivered a higher Faradaic efficiency than a silver‐free control, while maintaining stable chronopotentiometry and Faradaic efficiency over 8 h at a high current density of −55 mA cm^−2^.

**Figure 5:**
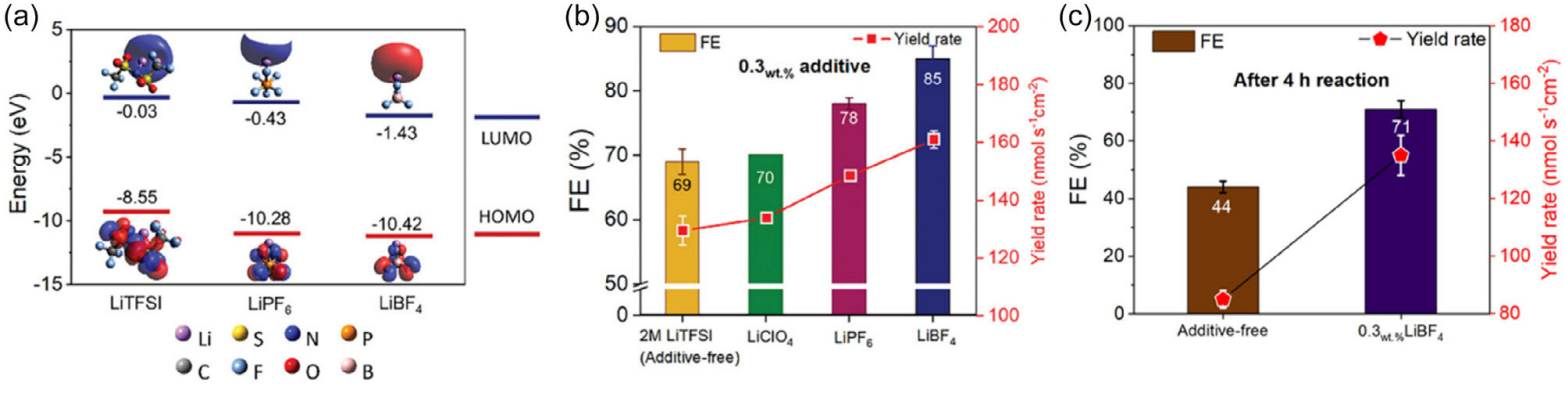
Fluoride additives affects Li‐NRR performance and stability. a) Energy level difference between LiTFSI, LiPF_6_, and LiBF_4_ calculated by G4MP2 calculation. b) Increase in ammonia yield rate and Faradaic efficiency with 0.3 wt% of various additives. c) Yield rate and Faradaic efficiency of two conditions (additive‐free, 0.3 wt% LiBF_4_) after 4 h reaction. Adapted with permission from ref. [60]. Copyright 2024, Wiley‐VCH GmbH.

Instead of adding specialized additives, some studies have pursued combining Li precursors to favorably tune SEI composition for better performance and stability. For instance, Shin et al. reported improvements in Li‐NRR by incorporating fluoride additives (LiBF_4_ or LiPF_6_) into a baseline LiTFSI–ethanol electrolyte.^[^ ^60^ ^]^ G4MP2 calculations showed that among LiBF_4_, LiPF_6_, and LiTFSI, LiBF_4_ possesses the lowest LUMO energy (see **Figure** **5**a), making it more readily reduced to LiF and thereby increasing the LiF fraction in the SEI. XPS and TOF‐SIMS analyses confirmed the elevated LiF content, and in line with other literature the LiTFSI  +  0.3 wt% LiBF_4_ formulation yielded superior performance and stability. In a 4 h reaction test, the LiBF_4_‐enhanced system exhibited a smaller drop in performance ([additive‐free] 69% → 44% versus [0.3 wt% LiBF_4_] 84% → 71%) (Figure 5b,c), and chronopotentiometry data similarly indicated a more stable working electrode potential.

### 3.3 Incorporation of Organic Additives in Li‐NRR

Organic chemicals can also be added to Li‐NRR systems to modulate SEI properties and improve overall performance. In some cases, these additives are directly incorporated into the SEI after undergoing decomposition under Li‐NRR conditions; in other instances, they function indirectly (analogous to solvents) by influencing Li‐ion solvation in the near the working electrode. Lim et al., for example, introduced dimethyl sulfide (DMS) into the electrolyte, thereby altering the physical structure of the SEI and enabling more stable Li‐NRR.^[^ ^61^ ^]^ In the Li‐NRR electrolyte, DMS is oxidized by LiClO_4_ to dimethyl sulfoxide (DMSO) and incorporated into the SEI, a process is confirmed by XPS and TOF‐SIMS analyses that revealed a decrease in LiClO_*n*_ content alongside increased Li_2_SO_4_ and Li_2_S fractions. This sulfur‐based SEI formed a uniform, net‐like structure that proved significantly more stable: whereas the working electrode potential in a reference electrolyte was more than doubled within 10 h under chronopotentiometric conditions, the DMS‐containing electrolyte was maintained with a steady potential for 20 h.

**Figure 6:**
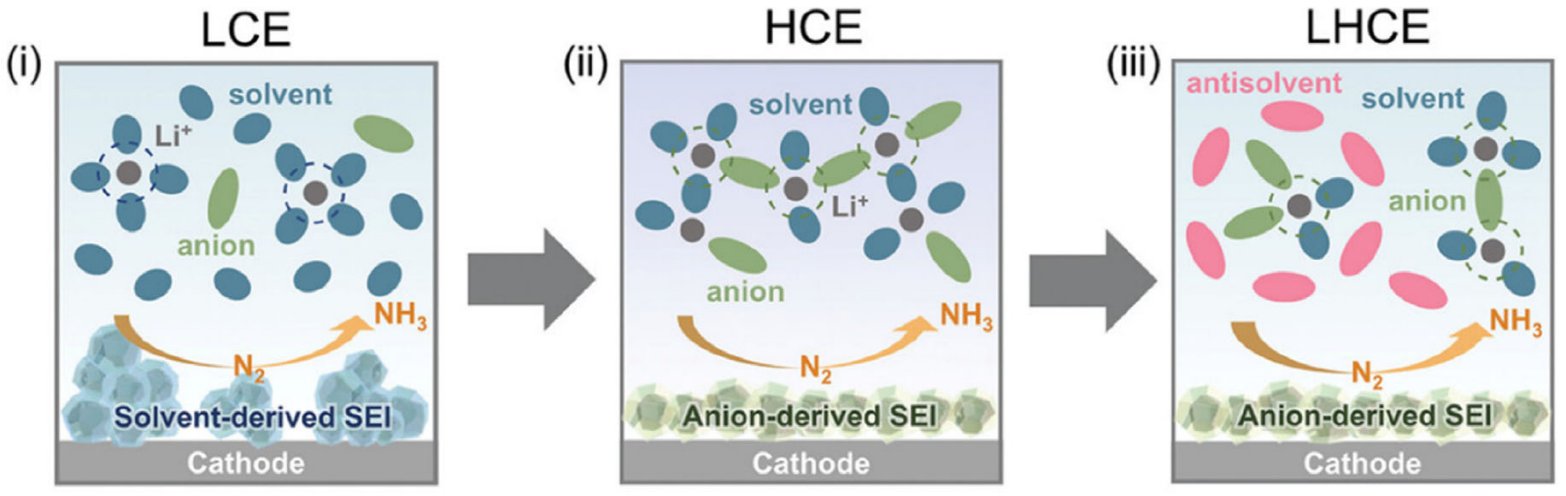
Schematic for incorporation of organic additives in Li‐NRR. The behavior of molecules around the SEI with LCE, HCE, and LHCE. Adapted with permission from ref. [62]. Copyright 2024, Wiley‐VCH GmbH.

Yun et al. were the first to introduce an antisolvent into a Li‐NRR electrolyte, using 1,1,2,2,‐tetrafluoroethyl‐2,2,3,3,‐tetrafluoropropyl ether (TTE).^[^ ^62^ ^]^ The resulting anion‐rich environment near the catalyst surface allowed a low‐concentration electrolyte (LCE) to emulate the benefits of a high‐concentration electrolyte (HCE)—notably, an anion‐rich SEI—while mitigating drawbacks of HCE like elevated viscosity and reduced cost efficiency (**Figure** **6**). XPS measurements confirmed that LiF peak intensities were higher in HCE relative to LCE and even higher under localized HCE (LHCE) conditions with TTE. Consistent with other findings, under LHCE conditions, beneficial features of LiF (improved ionic conductivity, suppressed electron conduction, minimized electrolyte decomposition, permeation of reactants, and thin, uniform SEI formation) enabled a stable, high‐efficiency Li‐NRR environment, with Faradaic efficiencies reaching ≈73%. Regarding stability, the working electrode potential remained constant over 20 h, and both the ammonia yield and Faradaic efficiency held steady for 12 h of operation.

Similar to this approach, the same group replaced THF with methylated THF (MTHF) to regulate conditions at the working electrode surface.^[^ ^63^ ^]^ Compared to THF, MTHF imposes additional steric hindrance on Li‐ion solvation, reducing Li^+^–solvent interactions and consequently allowing the TFSI^−^ anion to participate more prominently in Li^+^ solvation. During Li‐NRR, this phenomenon promotes the formation of an anion‐rich (inorganic) SEI. DFT calculations and Raman spectroscopy confirmed weakened Li^+^–THF interactions with MTHF addition, while SEM and XPS analyses revealed a higher LiF ratio in SEI and uniform SEI deposition, consistent with other studies. As a result, the MTHF‐based system achieved a maximum ammonia Faradaic efficiency of 65.8%, and its working electrode potential remained stable over a 10 h test.

## 4 Stabilizing Anodic Reaction in Li‐NRR

To maintain the stability of Li‐NRR systems, it is essential to manage the anodic reaction. As Li^+^ is reduced at the cathode, oxidation reactions must occur at the anode to balance the overall charge, which unavoidably leads to electrolyte decomposition such as THF oxidation or proton‐carrier oxidation.^[^ ^44^ ^]^ Furthermore, ammonia formed in the system also can be reoxidized at the anode, underlining the need for effective anodic control. If the anode reaction can be induced to minimize electrolyte decomposition, it becomes possible to design more stable, long‐term Li‐NRR systems.

Ideally, hydrogen consumed in the electrolyte for ammonia synthesis would be replenished at the anode. Toward this end, researchers have introduced H_2_ gas to induce the hydrogen oxidation reaction (HOR) at the anode, thereby allowing depleted proton carriers to be recharged with hydrogen.^[^ ^39^, ^40^, ^52^, ^64^, ^65^, ^66^ ^]^ Ensuring HOR at the anode is not only beneficial for regenerating proton carriers but also essential for the long‐term operation of Li‐NRR. In the absence of HOR, parasitic and irreversible oxidation of organic solvents becomes dominant, leading to the accumulation of byproducts and causing performance degradation in Li‐NRR. Accordingly, recent research has focused on enabling stable HOR at the anode. Efforts in this direction include (1) selecting proton carriers optimized for H_2_ recharging, (2) identifying HOR‐favorable catalysts, and (3) continuously feeding both N_2_ and H_2_ in a flow‐cell configuration to enable stable ammonia production. In the following sections, we review these approaches and their contributions to enhancing Li‐NRR stability.

### 4.1 H_2_ Addition/Regenerating Proton Carriers

Attempts to regenerate consumed proton carriers in one‐compartment closed Li‐NRR systems have been reported only in limited instances. For a proton carrier to be recharged instead of remaining a sacrificial species after ammonia synthesis, several requirements must be met: (1) the carrier anion left behind after ammonia formation must not readily participate in side reactions (i.e., it should remain stable and not decompose) and (2) upon introduction of a proton source (e.g., hydrogen gas), the carrier must be easily reprotonated to its original form.

To this end, Suryanto et al. proposed a phosphonium cation that reacts favorably with Li_3_N, but its anion form (ylide) can subsequently be regenerated by a weak acid.^[^ ^40^ ^]^ They demonstrated this concept in Li‐NRR by using a mixed‐gas feed of N_2_ and H_2_ (19.5 bar N_2_ + 0.5 bar H_2_), allowing the proton carrier to be recharged at the anode through its reaction with H_2_. Over a 20 h run, they maintained a stable ammonia synthesis at ≈69% Faradaic efficiency and 53 nmol s^−1^ cm^−2^. NMR analyses of the electrolyte before and after 20 h showed no significant side products and, notably, no changes in the phosphonium cation peaks, indicating that ammonia formation and proton‐carrier regeneration proceeded consistently in the presence of hydrogen gas. Moreover, because this result did not rely on a specialized anode catalyst or a gas diffusion electrode, it highlights the ionic liquid potential as a promising, sustainable proton carrier in various cell designs aimed at long‐term Li‐NRR.

Fu et al. provided the first direct confirmation of HOR induced by H_2_ addition in Li‐NRR. In contrast to earlier H_2_‐added Li‐NRR studies that relied on indirect evidence such as performance and stability comparisons or cyclic voltammetry, they used operando mass spectrometry during D_2_ oxidation experiments.^[^ ^39^ ^]^ Under D_2_ flow, the progressive decrease in H‐containing ammonia (NH_3_ and NH_2_D) accompanied by the increase in D‐containing ammonia (NHD_2_ and ND_3_) clearly demonstrated that HOR takes place in flow ammonia synthesis, with ethanol serving as a proton shuttle between NRR and HOR. A more detailed discussion of this work is provided in Chapter 4.3.

### 4.2 Anodes that Promoting HOR Reaction

**Figure 7:**
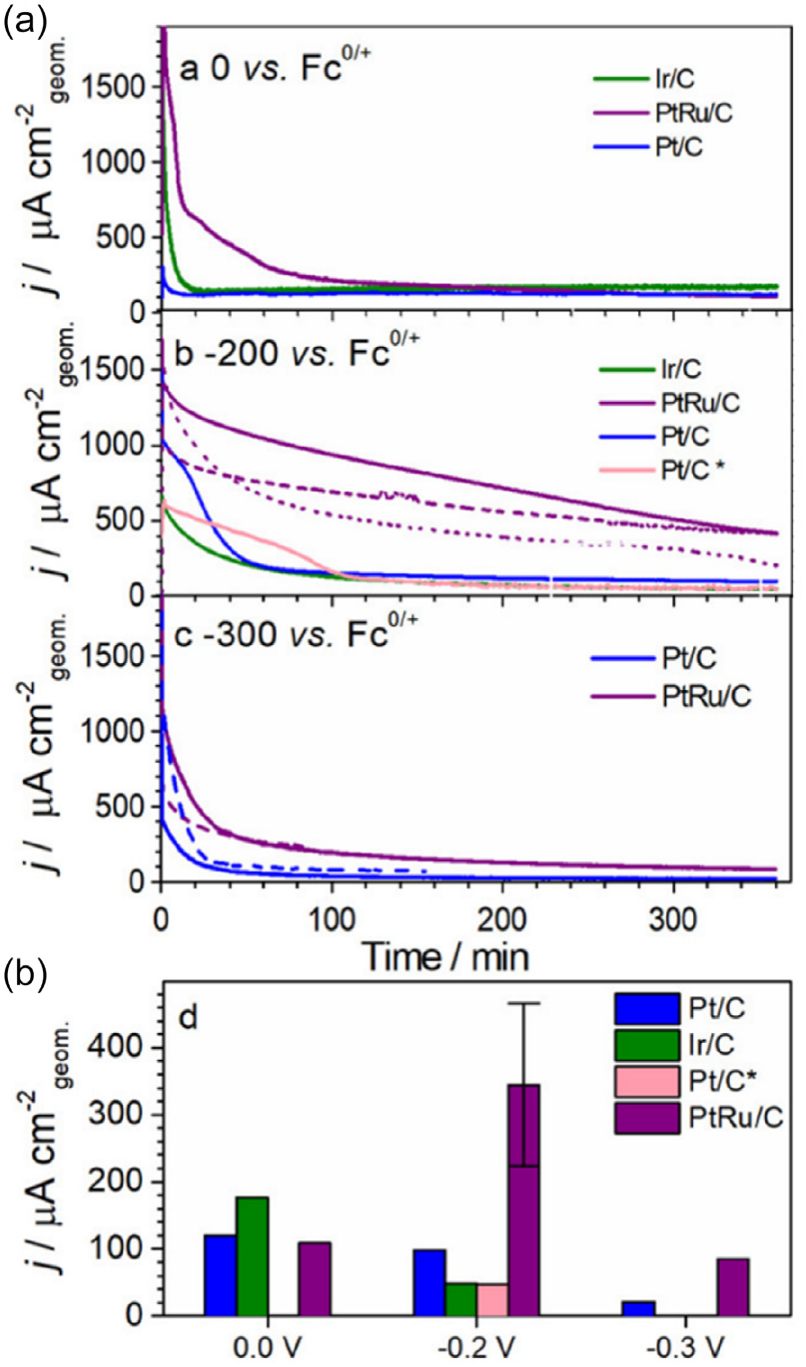
Chronoamperometry for H_2_ oxidation in H_2_‐saturated 0.1 M LiNTf_2_ THF solution. a) Chronoamperometry test for 360 min and b) current density measurement after 360 min with various catalysts (Ir/C, Pt/C, Pt/C with EtOH, PtRu/C) at three different applied potential. Adapted with permission from ref. [67]. Copyright 2022, American Chemical Society.

Hodgetts et al. proposed that selecting an appropriate anode is critical for effectively leveraging the regeneration of proton carriers in Li‐NRR systems supplied with hydrogen gas.^[^ ^67^ ^]^ In such setups, the anode must support sufficiently fast HORs, resist corrosion and surface contamination due to electrolyte adsorption, and exhibit robust durability under high potentials. Using a baseline THF‐based electrolyte with dissolved LiNTf_2_, they varied conditions (such as LiNTf_2_ concentration, water content, and ethanol addition) to evaluate multiple catalysts (Pt/C, Ru/C, PtRu/C, Au/C, Ir/C, Ni/C) for activity and poisoning susceptibility. Cyclic voltammetry tests indicated that PtRu/C offered the highest activity coupled with strong resistance to contamination (see **Figure** **7**a,b). This study thus provides a systematic approach for assessing HOR‐favorable catalysts in Li‐NRR environments and demonstrates that PtRu alloys may outperform pure Pt electrodes for stable Li‐NRR. Nevertheless, because research on anodic processes remains limited, the regeneration of proton carriers at the anode will likely emerge as an important focus in future Li‐NRR studies.

### 4.3 Flow Ammonia Synthesis

**Figure 8:**
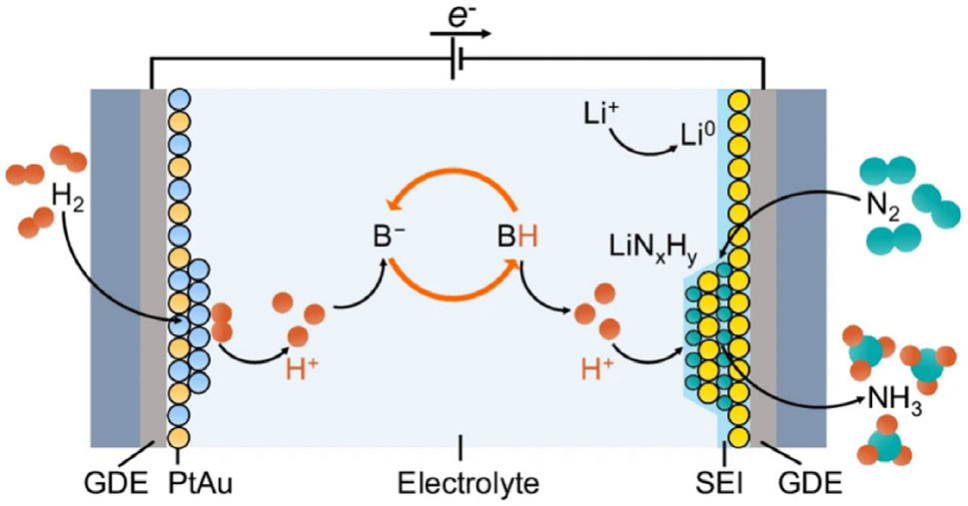
Li‐NRR in a continuous‐flow reactor (SSC GDE as the working electrode; PtAu/SSC GDE as the counter electrode). The proton carrier (BH in the scheme) supplies protons for NH_3_ synthesis, and its deprotonated form (B^−^ in the scheme) is regenerated at the counter electrode by reacting with hydrogen ions produced via HOR. Adapted with permission from ref. [52]. Copyright 2024, Springer Nature.

As efforts progressed to prevent depletion of proton carriers by introducing hydrogen gas into Li‐NRR systems, researchers began exploring flow‐cell configurations rather than conventional one‐compartment, three‐electrode closed cells. Flow cells offer the main advantage that their architecture directly supplies N_2_ and H_2_ through a gas diffusion electrode, establishing a gas–solid–liquid interface without requiring gas dissolution into the organic electrolyte (see **Figure** **8**). This enables Li‐NRR to overcome the severe N_2_ and H_2_ transport limitations typically encountered in single‐compartment cells, thereby facilitating the stable HOR discussed above and ultimately enabling long‐term, stable operation. In 2020, Lazouski et al. introduced the first flow‐cell‐based Li‐NRR architecture, using stainless steel cloth (SSC) as the working electrode and Pt‐plated Pt/SSC as the counter electrode.^[^ ^66^ ^]^ Their design purged N_2_ directly through the cathode and H_2_ through the anode, with a separator placed between the electrodes to prevent cross‐diffusion of reaction byproducts while allowing free movement of the proton carrier (ethanol and ethoxide). This pioneering flow‐cell approach successfully demonstrated Li‐NRR in a novel format, and it also proposed reactor coupled with water‐splitting reactor to supply electrochemically produced hydrogen gas.

Building on this foundational work, Fu et al. reported a flow‐cell‐based Li‐NRR system that delivered improved performance and substantially enhanced stability.^[^ ^39^ ^]^ In contrast to Lazouski et al.'s design, their system utilized a PtAu/SSC anode instead of Pt/SSC and did not physically separate the electrolyte compartments. Having identified that Pt in organic electrolytes is prone to contamination by intermediates—thereby losing active sites—they focused on identifying more robust counter electrode materials. Observing that PtAu alloys exhibit greater resistance to carbon monoxide (CO) poisoning than pure Pt, they used PtAu in a Li‐NRR flow cell and confirmed via computational and experimental assessments that the alloy had a lower affinity for organic species such as CO and THF. In practice, the PtAu anode exhibited stable potentials without appreciable electrolyte discoloration, indicating minimal side reactions and sustained catalytic activity. This improved flow Li‐NRR system to achieve a Faradaic efficiency of 61% and an energy efficiency of 13%, substantially surpassing the earlier benchmark. Moreover, under potential‐cycling conditions (1 min of Li deposition followed by 1 min of open‐circuit potential), the Faradaic efficiency remained consistently high over a 10 h run, evidencing excellent operational stability. Notably, their results also illustrate that ethanol, previously deemed unsuitable for recycling, can be continuously regenerated via H_2_ feeding, implying that ethanol can still be used in sustainable Li‐NRR designs. Subsequent work in the same system evaluated phenol and other proton carriers, demonstrating phenol particular advantages (as discussed in Chapter 2.3), and extended the analysis to lithium salts, concluding that LiBF_4_ represents a stable and efficient lithium precursor.^[^ ^52^ ^]^

## 5 Other Strategies for Improving Li‐NRR Stability

In addition to the three major strategies described above, there have been several noteworthy attempts aimed at improving stability in Li‐NRR systems. Numerous factors can be adjusted to influence both performance and stability—ranging from electrolyte composition and electrode materials to something as straightforward as altering how electrical energy is supplied.^[^ ^37^, ^41^, ^64^, ^68^, ^69^ ^]^

**Figure 9:**
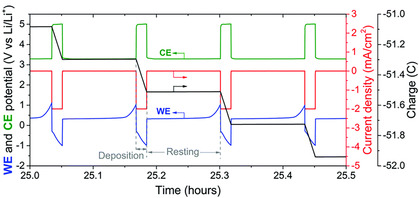
Potential cycling method for Li‐mediated ammonia production. Adapted with permission from ref. [37]. Copyright 2020, Royal Society of Chemistry.

For instance, Andersen et al. proposed a potential‐cycling method in which current is applied for 1 min, followed by 3–8 min of pause (no current), repeated cyclically (see **Figure** **9**).^[^ ^37^ ^]^ Under conventional continuous operation, the working electrode potential drifts to increasingly negative values in under an hour, whereas the potential‐cycling approach kept it remarkably stable for about 50 h. This method also boosted the ammonia synthesis efficiency from roughly 20 to 37%. The authors attributed these gains to reduced Li metal buildup, which otherwise leads to side reactions. By limiting the excessive deposition of Li, more portion of the deposited Li is directed toward lithium nitride and hence ammonia formation, enhancing both efficiency and stability. As this patterned power‐supply approach can be integrated with virtually any Li‐NRR research platform, it represents a potentially high‐impact strategy that could synergize with optimal electrolytes and electrode materials.

**Figure 10:**
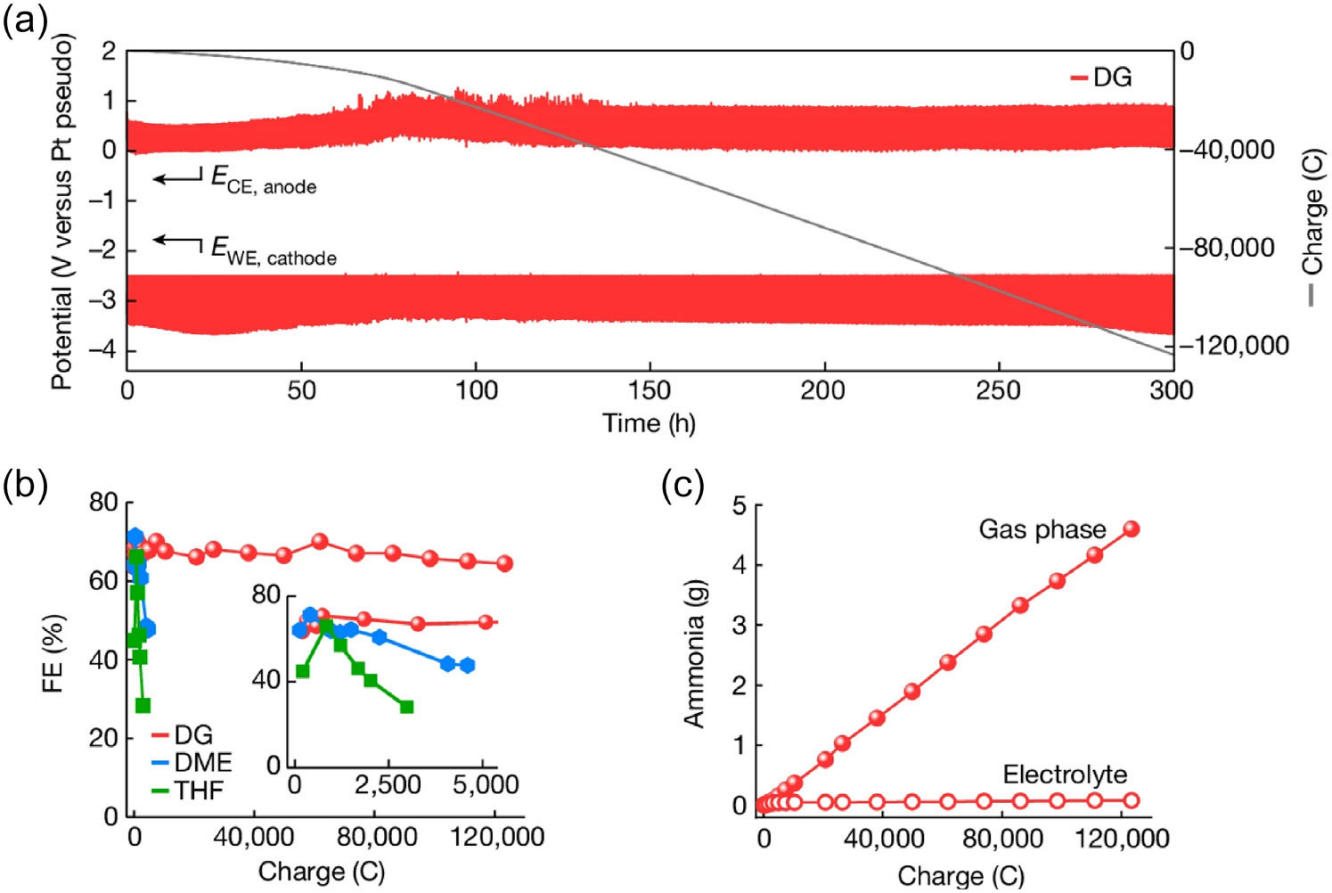
Long‐term continuous ammonia production under chain ether‐based electrolyte. a) The 300 h‐long ammonia synthesis with potential cycling method at −6 mA cm^−2^ using DG. b) Faradaic efficiency change for long‐term experiments using different solvents (THF, DME, DG). c) Amount of collected ammonia in the gas phase and dissolved in electrolyte. Adapted with permission from ref. [41]. Copyright 2024, Springer Nature.

Meanwhile, Li et al. investigated the viability of chain ethers as solvents for Li‐NRR, motivated by the limitations of THF.^[^ ^41^ ^]^ Although THF is the predominant solvent in nearly all Li‐NRR studies, its ring‐ether structure can undergo ring‐opening polymerization during reactions, causing electrolyte gelation that hinders extended operation. To circumvent this issue, Li et al. tested chain ethers dimethoxyethane (DME) and diethylene glycol dimethyl ether (DG) in a gas diffusion electrode‐based flow cell. In short experiments (1–4 h) with ethanol as the proton carrier, DME‐ and DG‐based systems yielded Faradaic efficiencies (81% and 76%, respectively) exceeding those achieved in THF (63%). SEM and NMR analyses revealed that, under DG, the SEI did not build up into thick, pore‐blocking layers on the GDE—unlike in THF. Instead, DG resisted ring‐opening side reactions (pre‐ and post‐reaction NMR spectra were virtually identical), forming only a thin, uniform SEI layer. Their stability tests were striking: under potential‐cycling conditions, the DG system maintained a steady working electrode potential, ammonia production (4.6 g cumulative NH_3_ over 300 h), and a Faradaic efficiency of 64% for an unprecedented 300 h (see **Figure** **10**a–c). This result marks a key milestone for moving beyond THF‐based systems, where issues like polymerization have long prevented the extended stability critical to commercializing Li‐NRR.

## 6 Summary and Outlook

In this review, we have focused on the recent progress in Li‐mediated nitrogen reduction (Li‐NRR) research, discussing various stability‐enhancing approaches and presenting representative examples. Although each strategy (choosing suitable proton carriers, devising favorable SEI environments, or controlling anodic reactions to reduce electrolyte decomposition and supply protons) has been driven by efforts to improve long‐term stability, they also offer clear benefits for ammonia production rates and Faradaic efficiency, making them key milestones toward Li‐NRR commercialization. Notable reports of Li‐NRR stability are summarized in **Table** **1**.

**Table 1:** Notable Li‐NRR stability reports.

Cell design	Cathode	Anode	Lithium precursor	Proton carrier	Solvent	Working electrode area [cm^2^]	Yield rate [nmol cm^−2 ^s^−1^]	FE [%]	Stability [h]	Ref.
Closed single cell	Mo foil	Pt mesh	LiClO_4_	EtOH	THF	1.8	0.7	37	50	[37]
Closed single cell	Ni wire	Cu wire	LiNTf_2_	EtOH	THF	0.05	150	99	96	[38]
Closed single cell	Cu disk	Pt coated Ti mesh	LiBF_4_	[P_6,6,6,14_]^+^	THF	0.012	53	69	20	[40]
Closed single cell	Cu disk	Pt coil	LiClO_4_	TBACl	THF	0.196	13.6	39.5	12	[49]
Closed single cell	Ni foil	Pt foil	LiTFSI	EtOH	THF/TTE	0.25	101	75	12	[62]
Flow cell	SSCa)	PtAu/SSC	LiBF_4_	EtOH	THF	25b)	–c)	61	10	[39]
Flow cell	SSC	PtAu/SSC	LiBF_4_	EtOH	DGd)	25b)	–c)	64	300	[41]

a) Stainless‐steel cloth;

b) Effective area of the GDE;

c) No definite yield rate reported;

d) Diethylene glycol dimethyl ether.

Nevertheless, analytical challenges continue to hinder Li‐NRR development. Fully characterizing the SEI on catalyst surfaces and capturing the state of the electrolyte both during and after reactions remains difficult. Because Li‐NRR must be conducted within an argon‐filled glovebox to exclude water and oxygen, transporting highly reactive Li samples to analytical instruments poses logistical hurdles, and in situ characterization is still in its infancy. Furthermore, many one‐compartment closed cell experiments are conducted under high pressures, making degassing necessary and risking physical damage to the SEI (e.g., from nitrogen bubbles). Despite these constraints, new analytical techniques such as in situ XPS, cryo‐EM, NMR, and TOF‐SIMS are emerging to provide more detailed insights into SEI formation and Li‐NRR mechanisms.^[^ ^44^, ^45^, ^53^, ^69^, ^70^, ^71^, ^72^, ^73^ ^]^ While early work (circa 2019–2021) primarily relied on XRD and XPS for qualitative compositional studies and SEM for morphological assessments—often with partial exposure to ambient conditions—the field is now increasingly adopting systematic characterization protocols and diverse analytical tools to guide more precise reaction strategies. This evolving landscape suggests that a comprehensive understanding of Li‐NRR mechanisms may be on the near horizon.

Another area requiring further attention is large‐scale experimentation. Because SEI properties on the working electrode surface critically affect both ammonia yield and system stability, uniform control of the SEI becomes more challenging as electrode and cell dimensions increase. Many one‐compartment Li‐NRR reports limit working‐electrode areas to ≈1 cm^2^, leading to comparatively low bulk ammonia production despite high rates per unit electrode area. In this regard, flow cells offer a promising solution for scaling up. Indeed, in the chain‐ether‐based electrolyte system reported by Li et al., a 36 cm^2^ working electrode was used to produce 4.6 g of ammonia over 300 h, demonstrating both longer operation and higher total yields under potential‐cycling conditions.^[^ ^41^ ^]^ Future work should thus focus on developing large‐area flow cells and achieving robust stability over extended durations to truly advance Li‐NRR toward practical applications.

Globally, interest in decarbonization and sustainable energy continues to rise, driving the importance of developing electrochemical pathways—potentially replacing Haber–Bosch—for green ammonia production. In just 5–8 years, Li‐NRR has seen remarkable growth, and extensive uncharted territory are also revealed, offering plenty scope for future breakthroughs. As this is one of the first reviews to systematically examine Li‐mediated NRR from the standpoint of stability enhancement, we hope it will serve as an inspiration for researchers aiming to create more robust and sustainable Li‐NRR systems. With continued advancements, Li‐mediated NRR may one day fully replace Haber–Bosch process entirely, realizing truly decentralized, carbon‐free, green ammonia.

## Conflict of Interest

The authors declare no conflict of interest.

## References

[1] USGS , Mineral Commodity Summaries 2021: U.S. Geological Survey 2021.

[2] S. H. So , S. J. Sung , S. J. Yang , C. R. Park , Where to go for the development of high‐performance H2 storage materials at ambient conditions? Electron. Mater. Lett. 2023, 19, 1.

[3] A. Valera‐Medina , H. Xiao , M. Owen‐Jones , W. I. F. David , P. J. Bowen , Ammonia for power, Prog. Energy Combust. Sci. 2018, 69, 63.

[4] A. Brunning , Environmental impact of industrial reactions, Chem. Eng. News 2019, 97, 23.

[5] F. Bird , A. Clarke , P. Davies , E. Surkovic , Ammonia: Zero‐Carbon Fertiliser, Fuelnd Energy Store, The Royal Society, London 2020, p. 1–40.

[6] D. R. MacFarlane , P. V. Cherepanov , J. Choi , B. H. R. Suryanto , R. Y. Hodgetts , J. M. Bakker , F. M. Ferrero Vallana , A. N. Simonov , A roadmap to the ammonia economy, Joule 2020, 4, 1186.

[7] M. Wang , M. A. Khan , I. Mohsin , J. Wicks , A. H. Ip , K. Z. Sumon , C. T. Dinh , E. H. Sargent , I. D. Gates , M. G. Kibria , Can sustainable ammonia synthesis pathways compete with fossil‐fuel based Haber‐Bosch processes? Energy Environ. Sci. 2021, 14, 2535.

[8] G. Qing , R. Ghazfar , S. T. Jackowski , F. Habibzadeh , M. M. Ashtiani , C. P. Chen , M. R. Smith , T. W. Hamann , Recent advances and challenges of electrocatalytic N_2_ reduction to ammonia, Chem. Rev. 2020, 120, 5437. 32459470 10.1021/acs.chemrev.9b00659

[9] C. Smith , A. K. Hill , L. Torrente‐Murciano , Current and future role of Haber–Bosch ammonia in a carbon‐free energy landscape, Energy Environ. Sci. 2020, 13, 331.

[10] M. Cong , M. Li , T. Liu , S. Hao , Z. Han , H. Xu , M. Guo , X. Ding , Y. Gao , Boosting nitrogen activation in tin oxides via heteroatom doping for efficient ammonia electrosynthesis, Chem. Eng. J. 2025, 519, 165405.

[11] Y. Xiong , Y. Wang , J. Zhou , F. Liu , F. Hao , Z. Fan , electrochemical nitrate reduction: ammonia synthesis and the beyond, Adv. Mater. 2024, 36, 2304021. 10.1002/adma.202304021 37294062

[12] N. Pirrone , S. Garcia‐Ballesteros , S. Hernández , F. Bella , Membrane/electrolyte interplay on ammonia motion inside a flow‐cell for electrochemical nitrogen and nitrate reduction, Electrochim. Acta 2024, 493, 144415.

[13] N. Pirrone , S. Garcia‐Ballesteros , J. Amici , M. Castellino , S. Hernández , F. Bella , Chemometrics‐boosted protocols for effortless evaluation of factors affecting the electrochemical nitrate reduction to ammonia, J. Energy Chem. 2025, 107, 599.

[14] P. García‐Negueroles , S. García‐Ballesteros , L. Santos‐Juanes , C. Sabater , M. A. Castillo , M. F. López‐Pérez , R. Vicente , A. M. Amat , A. Arques , Human like substances extracted from oil mill wastes in photo‐Fenton processes: Characterization, performance and toxicity assesment, J. Environ. Chem. Eng. 2021, 9, 106862.

[15] H. Huang , K. Peramaiah , K. W. Huang , Rethinking nitrate reduction: redirecting electrochemical efforts from ammonia to nitrogen for realistic environmental impacts, Energy Environ. Sci. 2024, 17, 2682.

[16] F. Rezaie , S. Læsaa , N. E. Sahin , J. Catalano , E. Dražević , Low‐temperature electrochemical ammonia synthesis: measurement reliability and comparison to Haber–Bosch in terms of energy efficienc, Energy Technol. 2023, 11, 2300410.

[17] J. Choi , B. H. R. Suryanto , D. Wang , H. L. Du , R. Y. Hodgetts , F. M. Ferrero Vallana , D. R. MacFarlane , A. N. Simonov , Identification and elimination of false positives in electrochemical nitrogen reduction studies, Nat. Commun. 2020, 11, 5546 33144566 10.1038/s41467-020-19130-z PMC7641139

[18] S. Z. Andersen , V. Čolić , S. Yang , J. A. Schwalbe , A. C. Nielander , J. M. McEnaney , K. Enemark‐Rasmussen , J. G. Baker , A. R. Singh , B. A. Rohr , M. J. Statt , S. J. Blair , S. Mezzavilla , J. Kibsgaard , P. C. K. Vesborg , M. Cargnello , S. F. Bent , T. F. Jaramillo , I. E. L. Stephens , J. K. Nørskov , I. Chorkendorff , A rigorous electrochemical ammonia synthesis protocol with quantitative isotope measurements, Nature 2019, 570, 504. 31117118 10.1038/s41586-019-1260-x

[19] B. H. R. Suryanto , H. L. Du , D. Wang , J. Chen , A. N. Simonov , D. R. MacFarlane , Challenges and prospects in the catalysis of electroreduction of nitrogen to ammonia, Nat. Catal. 2019, 2, 290.

[20] S. Maheshwari , M. J. Janik , Kinetics of Li‐mediated N_2_ electroreduction, Joule 2019, 3, 915.

[21] N. Lazouski , Z. J. Schiffer , K. Williams , K. Manthiram , Understanding continuous lithium‐mediated electrochemical nitrogen reduction, Joule 2019, 3, 1127.

[22] J. A. Schwalbe , M. J. Statt , C. Chosy , A. R. Singh , B. A. Rohr , A. C. Nielander , S. Z. Andersen , J. M. McEnaney , J. G. Baker , T. F. Jaramillo , J. K. Norskov , M. Cargnello , A combined theory‐experiment analysis of the surface species in lithium‐mediated NH_3_ electrosynthesis, ChemElectroChem 2020, 7, 1542.

[23] S. Li , X. Fu , J. K. Nørskov , I. Chorkendorff , Towards sustainable metal‐mediated ammonia electrosynthesis, Nat. Energy 2024, 9, 1344.

[24] W. Chang , A. Jain , F. Rezaie , K. Manthiram , Lithium‐mediated nitrogen reduction to ammonia via the catalytic solid–electrolyte interphase, Nat. Catal. 2024, 7, 231.

[25] M. S. Iqbal , Y. Ruan , R. Iftikhar , F. Z. Khan , W. Li , L. Hao , A. W. Robertson , G. Percoco , Z. Sun , Lithium‐mediated electrochemical dinitrogen reduction reaction, Ind. Chem. Mater. 2023, 1, 563.

[26] M. I. Ahmed , A. Assafiri , D. B. Hibbert , C. Zhao , Li‐mediated electrochemical nitrogen fixation: Key advances and future perspectives, Small 2023, 19, 2305616. 10.1002/smll.202305616 37635122

[27] M. Jiang , X. Chen , F. Chen , M. Wang , X. Luo , Y. He , C. Wu , L. Zhang , X. Li , X. Liao , Z. Jiang , Z. Jin , Effective N_2_ activation strategies for electrochemical ammonia synthesis, Chem 2025, 11, 102441.

[28] A. Mangini , L. Fagiolari , A. Sacchetti , A. Garbujo , P. Biasi , F. Bella , Lithium‐mediated nitrogen reduction for ammonia synthesis: reviewing the gap between continuous electrolytic cells and stepwise processes through galvanic Li—N_2_ cells, Adv. Energy Mater. 2024, 14, 2400076.

[29] X. Fu , Lithium‐mediated nitrogen reduction for electrochemical ammonia synthesis: From batch to flow reactor, Mater. Today Catal. 2023, 3, 100031.

[30] C. Burdis , S. Zamany Andersen , J. Barrio , M. Titirici , I. E. L. Stephens , M. Saccoccio , Recent advances in metal‐mediated electrochemical ammonia synthesis towards commercialization, Curr. Opin. Green Sustain. Chem. 2024, 50, 100964.

[31] C. K. Klein , K. Manthiram , Sustainable ammonia synthesis: Just around the corner? Joule 2022, 6, 1971.

[32] M. A. Yusov , K. Manthiram , Beyond lithium for sustainable ammonia synthesis, Nat. Mater. 2024, 23, 31. 38110514 10.1038/s41563-023-01747-2

[33] M. C. Hatzell , A decade of electrochemical ammonia synthesis, ACS Energy Lett. 2022, 7, 4132.

[34] F. Fichter , P. Girard , H. Erlenmeyer , Elektrolytisehe bindung von komprimiertem stiekstoff bei gewohnlieher temperature, Helv. Chim. Acta 1930, 13, 1228.

[35] A. Tsuneto , A. Kudo , T. Sakata , Efficient electrochemical reduction of N_2_ to NH_3_ catalyzed by lithium, Chem. Lett. 1993, 22, 851.

[36] A. Tsuneto , A. Kudo , T. Sakata , Lithium‐mediated electrochemical reduction of high pressure N2 to NH3, J. Electroanal. Chem. 1994, 367, 183.

[37] S. Z. Andersen , M. J. Statt , V. J. Bukas , S. G. Shapel , J. B. Pedersen , K. Krempl , M. Saccoccio , D. Chakraborty , J. Kibsgaard , P. C. K. Vesborg , J. Nørskov , I. Chorkendorff , Increasing stability, efficiency, and fundamental understanding of lithium‐mediated electrochemical nitrogen reduction, Energy Environ. Sci. 2020, 13, 4291.

[38] H. L. Du , M. Chatti , R. Y. Hodgetts , P. V. Cherepanov , C. K. Nguyen , K. Matuszek , D. R. MacFarlane , A. N. Simonov , Electroreduction of nitrogen with almost 100% current‐to‐ammonia efficiency, Nature 2022, 609, 722. 35868345 10.1038/s41586-022-05108-y

[39] X. Fu , J. B. Pedersen , Y. Zhou , M. Saccoccio , S. Li , R. Sažinas , K. Li , S. Z. Andersen , A. Xu , N. H. Deissler , J. B. Valbæk Mygind , C. Wei , J. Kibsgaard , P. C. K. Vesborg , J. K. Nørskov , I. Chorkendorff , Continuous‐flow electrosynthesis of ammonia by nitrogen reduction and hydrogen oxidation Science 2023, 379, 707. 36795804 10.1126/science.adf4403

[40] B. H. R. Suryanto , K. Matuszek , J. Choi , R. Y. Hodgetts , H. L. Du , J. M. Bakker , C. S. M. Kang , P. V. Cherepanov , A. N. Simonov , D. R. MacFarlane , Nitrogen reduction to ammonia at high efficiency and rates based on a phosphonium proton shuttle, Science 2021, 372, 1187. 34112690 10.1126/science.abg2371

[41] S. Li , Y. Zhou , X. Fu , J. B. Pedersen , M. Saccoccio , S. Z. Andersen , K. Enemark‐Rasmussen , P. J. Kempen , C. D. Damsgaard , A. Xu , R. Sažinas , J. B. V. Mygind , N. H. Deissler , J. Kibsgaard , P. C. K. Vesborg , J. K. Nørskov , I. Chorkendorff , Long‐term continuous ammonia electrosynthesis, Nature 2024, 629, 92. 38503346 10.1038/s41586-024-07276-5

[42] A. Mangini , J. B. V. Mygind , S. G. Ballesteros , A. Pedico , M. Armandi , I. Chorkendorff , F. Bella , Multivariate approaches boosting lithium‐mediated ammonia electrosynthesis in different electrolytes, Angew. Chem. Int. Ed. 2025, 64, e202416027. 10.1002/anie.202416027 PMC11833281 39824767

[43] L. F. Gao , Y. Cao , C. Wang , X. W. Yu , W. B. Li , Y. Zhou , B. Wang , Y. F. Yao , C. P. Wu , W. J. Luo , Z. G. Zou , Domino effect: Gold electrocatalyzing lithium reduction to accelerate nitrogen fixation, Angew. Chem. Int. Ed. 2021, 60, 5257. 10.1002/anie.202015496 33251671

[44] H. L. Du , K. Matuszek , R. Y. Hodgetts , K. Ngoc Dinh , P. V. Cherepanov , J. M. Bakker , D. R. MacFarlane , A. N. Simonov , The chemistry of proton carriers in high‐performance lithium‐mediated ammonia electrosynthesis, Energy Environ. Sci. 2023, 16, 1082.

[45] K. Steinberg , X. Yuan , C. K. Klein , N. Lazouski , M. Mecklenburg , K. Manthiram , Y. Li , Imaging of nitrogen fixation at lithium solid electrolyte interphases via cryo‐electron microscopy, Nat. Energy 2023, 8, 138.

[46] D. Krishnamurthy , N. Lazouski , M. L. Gala , K. Manthiram , V. Viswanathan , Closed‐loop electrolyte design for lithium‐mediated ammonia synthesis, ACS Cent. Sci. 2021, 7, 2073. 34963899 10.1021/acscentsci.1c01151 PMC8704027

[47] N. Lazouski , K. J. Steinberg , M. L. Gala , D. Krishnamurthy , V. Viswanathan , K. Manthiram , Proton donors induce a differential transport effect for selectivity toward ammonia in lithium‐mediated nitrogen reduction, ACS Catal. 2022, 12, 5197.

[48] N. T. Nguyen , L. A. O’Dell , K. N. Dinh , R. Y. Hodgetts , C. K. Nguyen , K. Banerjee , D. T. H. Truong , J. M. Bakker , A. McKay , D. R. MacFarlane , H. L. Du , A. N. Simonov , Nitrogen electroreduction to ammonia with phosphonium proton shuttles: Mass‐transport versus electrode surface chemistry effects, Chem 2024, 10, 3622.

[49] S. Yang , J. Chu , J. Park , H. Kim , B. Shin , Enhancement of lithium‐mediated nitrogen reduction by modifying center atom of tetraalkyl‐type ionic liquids, Angew. Chem. Int. Ed. 2024, 63, e202411909. 10.1002/anie.202411909 39183595

[50] J. Hyung Kim , J. E. Cha , H. K. Ju , Y. W. Choi , J. Baek , J. Georg Albers , J. Shim , S. Hyung Kim , K. Lee , H. Chul Yoon , Utilizing water as a proton source for sustainable Li‐mediated electrochemical ammonia synthesis, Chem. Eng. J. 2024, 497, 154644.

[51] J. Bjarke Valbæk Mygind , J. B. Pedersen , K. Li , N. H. Deissler , M. Saccoccio , X. Fu , S. Li , R. Sažinas , S. Z. Andersen , K. Enemark‐Rasmussen , P. C. K. Vesborg , J. Doganli‐Kibsgaard , I. Chorkendorff , Is ethanol essential for the lithium‐mediated nitrogen reduction reaction? ChemSusChem 2023, 16, e202301011. 37681646 10.1002/cssc.202301011

[52] X. Fu , A. Xu , J. B. Pedersen , S. Li , R. Sažinas , Y. Zhou , S. Z. Andersen , M. Saccoccio , N. H. Deissler , J. B. V. Mygind , J. Kibsgaard , P. C. K. Vesborg , J. K. Nørskov , I. Chorkendorff , Phenol as proton shuttle and buffer for lithium‐mediated ammonia electrosynthesis, Nat. Commun. 2024, 15, 2417. 38499554 10.1038/s41467-024-46803-w PMC10948763

[53] N. H. Deissler , J. B. V. Mygind , K. Li , V. A. Niemann , P. Benedek , V. Vinci , S. Li , X. Fu , P. C. K. Vesborg , T. F. Jaramillo , J. Kibsgaard , J. Drnec , I. Chorkendorff , Operando investigations of the solid electrolyte interphase in the lithium mediated nitrogen reduction reaction, Energy Environ. Sci. 2024, 17, 3482.

[54] E. Wang , S. Dey , T. Liu , S. Menkin , C. P. Grey , Effects of atmospheric gases on Li metal cyclability and solid‐electrolyte interphase formation, ACS Energy Lett. 2020, 5, 1088. 32300662 10.1021/acsenergylett.0c00257 PMC7155172

[55] K. Li , S. Z. Andersen , M. J. Statt , M. Saccoccio , V. J. Bukas , K. Krempl , R. Sažinas , J. B. Pedersen , V. Shadravan , Y. Zhou , D. Chakraborty , J. Kibsgaard , P. C. K. Vesborg , J. K. Nørskov , I. Chorkendorff , Enhancement of lithium‐mediated ammonia synthesis by addition of oxygen, Science 2021, 374, 1593. 34941415 10.1126/science.abl4300

[56] O. J. Kim , Y. H. Cho , J. J. Kang , Y. S. Yu , C. Kim , G. Y. Yun , Exploration about the electrolyte system of Li‐ion batteries for the wide temperature range operation, Electron. Mater. Lett. 2024, 10.1007/s13391-024-00488-x.

[57] R. Sažinas , K. Li , S. Z. Andersen , M. Saccoccio , S. Li , J. B. Pedersen , J. Kibsgaard , P. C. K. Vesborg , D. Chakraborty , I. Chorkendorff , Oxygen‐enhanced chemical stability of lithium‐mediated electrochemical ammonia synthesis, J. Phys. Chem. Lett. 2022, 13, 4605. 35588323 10.1021/acs.jpclett.2c00768 PMC9150109

[58] M. Spry , O. Westhead , R. Tort , B. Moss , Y. Katayama , M. M. Titirici , I. E. L. Stephens , A. Bagger , water increases the faradaic selectivity of Li‐ mediated nitrogen reduction, ACS Energy Lett. 2023, 8, 1230. 36816776 10.1021/acsenergylett.2c02792 PMC9926485

[59] Y. Jeon , D. Shin , K. Yong , Y. J. Hwang , Enhancing ammonia production by Ag incorporation in Li‐mediated nitrogen reduction reactions, ACS Energy Lett., 2024, 9, 4147.

[60] D. Shin , Y. Jeon , V. T. Nguyen , S. Kang , Y. Hong , C. Lim , K. Yong , H. Shin , Y. J. Hwang , Insight into fluoride additives to enhance ammonia production from lithium‐mediated electrochemical nitrogen reduction reaction, Small 2024, 20, 2404525. 10.1002/smll.202404525 38984768

[61] C. Lim , D. Kim , M. Kim , H. Yun , D. Shin , Y. J. Hwang , H. Shin , K. Yong , Effect of sulfur‐derived solid electrolyte interphase on Li‐mediated nitrogen reduction, ACS Energy Lett. 2023, 8, 4875.

[62] H. Yun , C. Lim , M. Kwon , D. Lee , Y. Yun , D. H. Seo , K. Yong , Localized high‐concentration electrolyte in li‐mediated nitrogen reduction for ammonia synthesis Adv. Mater. 2024, 36, 2408280. 39434486 10.1002/adma.202408280 PMC11619219

[63] Y. Han , C. Lim , Y. Kim , H. Baek , S. Jeon , J. W. Han , K. Yong , Steric hindrance‐derived Li^+^ solvation to enhance lithium‐mediated nitrogen reduction, ACS Energy Lett. 2024, 9, 5509.

[64] X. Fu , S. Li , N. H. Deissler , J. B. V. Mygind , J. Kibsgaard , I. Chorkendorff , Effect of lithium salt on lithium‐mediated ammonia synthesis, ACS Energy Lett. 2024, 9, 3790.

[65] X. Cai , Z. Shadike , X. Cai , X. Li , L. Luo , L. An , J. Yin , G. Wei , F. Yang , S. Shen , J. Zhang , Membrane electrode assembly design for lithium‐ mediated electrochemical nitrogen reduction, Energy Environ. Sci. 2023, 16, 3063.

[66] N. Lazouski , M. Chung , K. Williams , M. L. Gala , K. Manthiram , Non‐aqueous gas diffusion electrodes for rapid ammonia synthesis from nitrogen and water‐splitting‐derived hydrogen, Nat. Catal. 2020, 3, 463.

[67] R. Y. Hodgetts , H. L. Du , T. D. Nguyen , D. Macfarlane , A. N. Simonov , Electrocatalytic oxidation of hydrogen as an anode reaction for the Li‐mediated N2 reduction to ammonia, ACS Catal. 2022, 12, 5231.

[68] D. Shin , A. Choi , D. Y. Han , G. Bak , S. Yoo , Y. Jeon , S. Park , Y. J. Hwang , Enhancing lithium‐mediated nitrogen reduction with porous polymer fibers featuring lithium‐ion affinity, Adv. Funct. Mater. 2025, 35, 2416484.

[69] S. Li , Y. Zhou , K. Li , M. Saccoccio , R. Sažinas , S. Z. Andersen , J. B. Pedersen , X. Fu , V. Shadravan , D. Chakraborty , J. Kibsgaard , P. C. K. Vesborg , J. K. Nørskov , I. Chorkendorff , Electrosynthesis of ammonia with high selectivity and high rates via engineering of the solid‐electrolyte interphase, Joule 2022, 6, 2083. 36188748 10.1016/j.joule.2022.07.009 PMC9511958

[70] R. Luo , A. Bergljót Gunnarsdóttir , E. W. Zhao , Direct in situ NMR observation of lithium plating, corrosion, nitridation and protonolysis for ammonia synthesis Ruipeng, Chemrxiv preprint 2024, 10.26434/chemrxiv-2024-cpf4j.

[71] E. J. McShane , V. A. Niemann , P. Benedek , X. Fu , A. C. Nielander , I. Chorkendorff , T. F. Jaramillo , M. Cargnello , Quantifying influence of the solid‐electrolyte interphase in ammonia electrosynthesis, ACS Energy Lett. 2023, 8, 4024.

[72] S. J. Blair , M. Doucet , V. A. Niemann , K. H. Stone , M. E. Kreider , J. F. Browning , C. E. Halbert , H. Wang , P. Benedek , E. J. McShane , A. C. Nielander , A. Gallo , T. F. Jaramillo , Combined, time‐resolved, in situ neutron reflectometry and X‐ray diffraction analysis of dynamic SEI formation during electrochemical N_2_ reduction, Energy Environ. Sci. 2023, 16, 3391.

[73] S. J. Blair , M. Doucet , J. F. Browning , K. Stone , H. Wang , C. Halbert , J. Avilés Acosta , J. A. Zamora Zeledón , A. C. Nielander , A. Gallo , T. F. Jaramillo , Lithium‐mediated electrochemical nitrogen reduction: tracking electrode−electrolyte interfaces via time−resolved neutron reflectometry, ACS Energy Lett. 2022, 7, 1939.

